# The “care” protocol: outcome of medication overuse headache in a three year follow-up study

**DOI:** 10.1186/1129-2377-14-S1-P175

**Published:** 2013-02-21

**Authors:** G Sances, N Ghiotto, F Galli, M Allena, A Frustaci, E Guaschino, C Tassorelli, G Nappi

**Affiliations:** 1 Headache Science Centre of the IRCCS ‘National Institute of Neurology C. Mondino’ Foundation, Pavia, Italy; 2 Clinical and Molecular Epidemiology, IRCCS San Raffaele Pisana, Rome, Italy

## Introduction

Medication Overuse Headache (MOH) has become one of the major challenges in headache management. The main aim of the present study was to evaluate the factors associated with a negative outcome in a three-year follow-up of subjects diagnosed with MOH.

## Methods

All consecutive patients with MOH entering, for the first time, the centre’s inpatient detoxification program were analyzed in a prospective, non randomized way. They were enrolled as outpatients and gave their verbal informed consent to undergo the protocol (inpatient detoxification and three follow-up visits in the first year, then six-monthly clinical controls). The diagnosis of MOH was made according to the revised-ICHD-II criteria [1]. All the participants were assessed using an ad hoc patient’s record form. Variables analyzed as possible predictors were: gender, age, socio-demographic characteristics, alcohol/coffee/smoking habits, positive family history for drug abuse and/or headache, past medical history, primary headache type, type, duration and quantification of drug overuse and duration of chronic headache. Categorical variables were analyzed with the Chi-square test. For quantitative variables, statistical differences were analyzed with ANOVA. Odds Ratios (ORs) were calculated for dichotomous outcomes as well.

## Findings

One-hundred-fifty patients completed the 3-year follow-up (79.3% females, age 46.40±11.31): 13 patients never stopped overuse (Group A), 38 patients stopped drug overuse, but relapsed at least once (Group B) and 99 patients never relapsed (Group C). Patients in Group A differed from B+C groups because they were more frequently single (OR 0.134;p=0.007) and unemployed (OR=3.273;p=0.04), they took a higher number of acute drugs (p<0.001) and used less frequently coffee (OR 3.273;p=0.044).

## Conclusion

The outcome of disease in this group of MOH patients was influenced negatively by the severity of overuse (and possibly of the disease) and by specific socio-economic conditions. Other factors that emerged as possible modifiers of outcome were voluptuary habits.
